# Biographical Feature: Raymond M. Welsh, Jr. (1945–2024)—a life in viral immunology, immune regulation, and scientific mentorship

**DOI:** 10.1128/jvi.00663-26

**Published:** 2026-06-17

**Authors:** Randy R. Brutkiewicz, Michael Brehm, Heather D. Marshall, Carey L. O’Donnell, Steven M. Varga, Stephen N. Waggoner

**Affiliations:** 1 Department of Microbiology and Immunology, Indiana University School of Medicine 734638, Indianapolis, Indiana, USA; 2 Diabetes Center of Excellence, Program in Molecular Medicine, The University of Massachusetts Chan Medical School 378174, Worcester, Massachusetts, USA; 3 DynaMed, EBSCO 650769 , Ipswich, Massachusetts, USA; 4 Department of Infectious Diseases, St. Jude Children's Research Hospital 5417, Memphis, Tennessee, USA; 5 Department of Pediatrics, Cincinnati Children’s Hospital Medical Center, University of Cincinnati College of Medicine 170291, Cincinnati, Ohio, USA; Dartmouth College Geisel School of Medicine, Hanover, New Hampshire, USA

## TEXT

**Figure:**
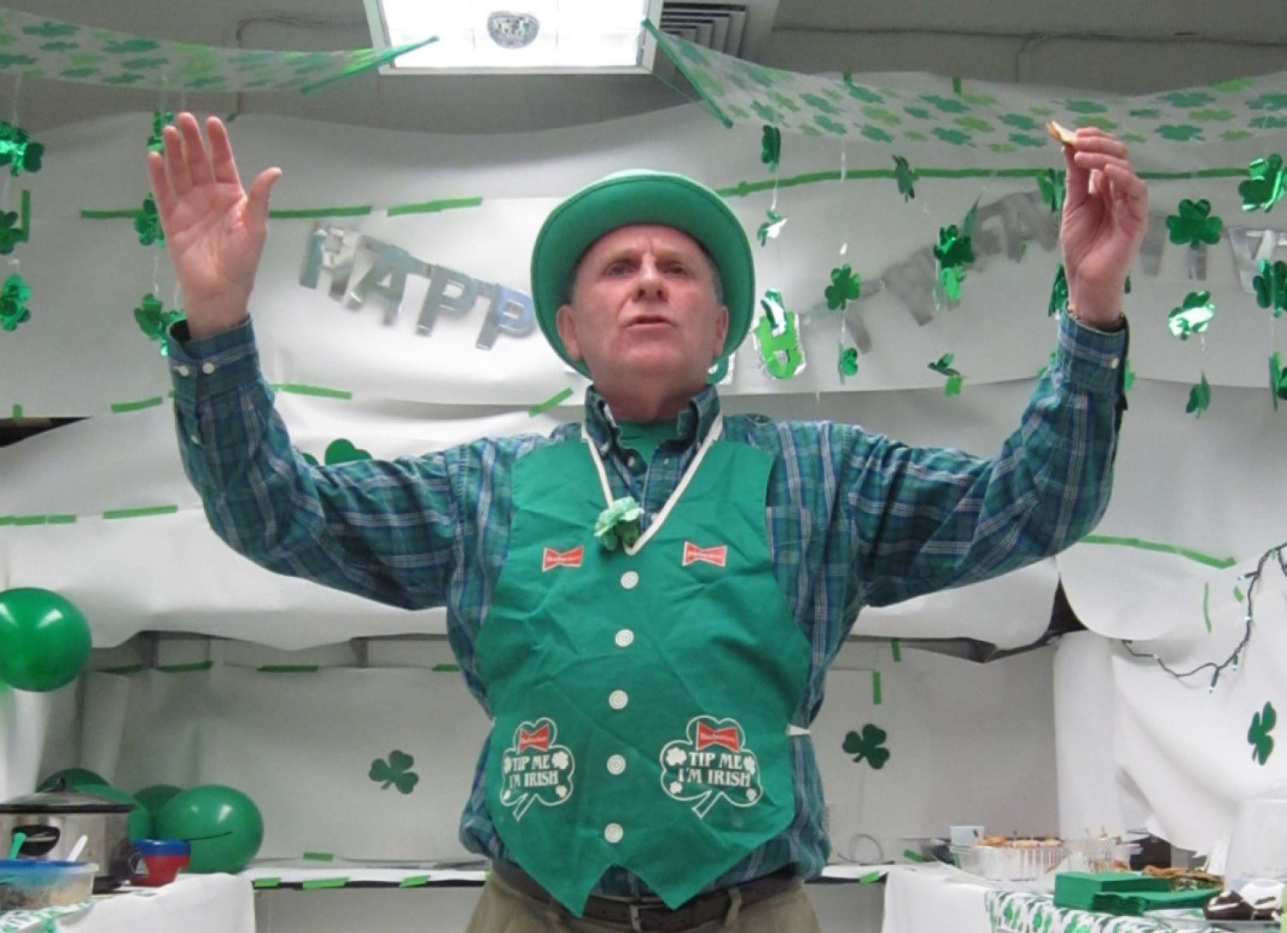


The history of viral immunology in the late 20th and early 21st centuries cannot be written without sustained attention to the work of our mentor and friend, Dr. Raymond (Ray) M. Welsh, Jr. Over more than four decades, Ray helped define how virologists and immunologists conceptualize host–virus interactions, immune regulation, and immunological memory (1, 2). His scientific contributions reshaped understanding of natural killer (NK) cells, virus-induced immunopathology, heterologous immunity, and the dynamic nature of immune memory (2–5). At the same time, his influence extended far beyond his own experiments through a remarkable record of mentorship, service, and community building.

Ray’s career unfolded during a period of conceptual upheaval in immunology, as classical distinctions between innate and adaptive immunity, specificity and cross-reactivity, and protection and pathology were repeatedly challenged. Ray was not merely a participant in these debates; he was one of the scientists who forced the field to confront uncomfortable complexities. His work demonstrated that immune responses to viral infection are rarely linear, uniformly beneficial, or immunologically isolated. Instead, they are shaped by prior immune history, by competition among lymphocyte populations, and by regulatory circuits that can protect the host at one moment and contribute to pathology at another.

Equally important was Ray’s role as a mentor. We former trainees consistently recall an environment that emphasized independence, intellectual rigor, and personal accountability. Many of us went on to become leaders in academia, biotechnology, industry, and government research, carrying both the scientific frameworks and mentoring philosophy that characterized the Welsh laboratory.

This biographical feature traces Ray’s life and career, integrating his scientific achievements with reflections from those of us who trained with him. Together, these perspectives reveal a scientist whose legacy resides not only in an extraordinary body of work, but also in a scientific culture he helped create.

Raymond M. Welsh, Jr. was born on 28 December 1945 in Montague City, Massachusetts. His early education coincided with a period of rapid expansion in American biomedical science, driven by postwar investment in research and higher education. Ray pursued undergraduate training in microbiology at the University of Massachusetts Amherst, earning his Bachelor of Science degree in 1967. This early exposure to microbiology provided the conceptual foundation for a career that would integrate virology, immunology, and pathology.

Following brief additional study in microbiology at the University of Miami, Ray undertook graduate training at Rensselaer Polytechnic Institute before returning to UMass Amherst, where he completed his Ph.D. in microbiology and virology in 1972. His doctoral work focused on lymphocytic choriomeningitis virus (LCMV), a model arenavirus that would become central to his scientific identity (6). Even in this early work, Ray demonstrated a distinctive interest in regulatory phenomena—defective interfering particles, persistent infection, and the host’s response to chronic viral infection (6).

Ray’s postdoctoral training further shaped his scientific trajectory. He served as a postdoctoral research associate and a visiting assistant professor at the University of Kansas, where he gained experience teaching and refining his independent research vision. He then moved to the Scripps Clinic and Research Foundation in La Jolla, California, first as a research fellow and later as an assistant member in the Department of Immunopathology. At Scripps, Ray worked in an intellectually vibrant environment that emphasized the immunological consequences of viral infection, complement-mediated viral neutralization, and immune-mediated tissue injury. These experiences crystallized his lifelong focus on the intersection of virology and immunology.

In 1980, Ray was recruited to the University of Massachusetts Medical School (UMMS) in Worcester, Massachusetts. Appointed initially as an associate professor in the Departments of Pathology and Molecular Genetics and Microbiology, he was promoted to full professor in 1985. UMMS would remain his academic home for the next 35 years.

From the outset, the Welsh laboratory distinguished itself through both productivity and intellectual ambition. Ray established a research program that was conceptually integrated yet experimentally diverse, using viral infection as a lens through which to examine immune regulation. While LCMV remained a central model, the laboratory expanded to include other viral systems, transplantation models, and tumor-associated viral infections.

Ray’s leadership extended beyond his laboratory. He served multiple terms as chair or vice-chair of the Interdepartmental Immunology and Virology Program at UMMS and was deeply involved in graduate education, curriculum development, and institutional governance. His role as principal investigator on a long-standing NIH T32 training grant reflected his commitment to fostering interdisciplinary training in immunology and virology.

Among Ray’s most enduring scientific contributions was his work on natural killer cells. When he began studying NK cells in the 1970s and early 1980s, these cells were poorly understood and often regarded as immunological curiosities. Through a series of rigorous *in vivo* studies, Ray demonstrated that NK cells play decisive roles in the control of viral infections (3–5), often acting independently of classical adaptive immune mechanisms.

Ray’s laboratory elucidated the kinetics of NK cell induction during viral infection, their trafficking to infected tissues, and their interactions with interferons and cytokines (4, 5). Importantly, his work showed that NK cell activity could be both protective and pathological depending on context. These findings challenged simplistic narratives of innate immunity as uniformly beneficial and underscored the need to understand immune responses as regulated systems.

One of Ray’s key conceptual advances was the recognition that NK cells function as regulators of adaptive immunity (7). Rather than acting solely as early antiviral effectors, NK cells could shape the magnitude and quality of T cell responses, influencing viral clearance, immune memory, and immunopathology. This idea, later articulated as NK cells acting as “rheostats” of immune responses (7), profoundly influenced subsequent work in viral immunology.

Throughout his career, Ray returned repeatedly to LCMV as an experimental system (8). His sustained engagement with this model virus allowed his laboratory to explore immune regulation with exceptional depth. His work on LCMV addressed fundamental questions about cytotoxic T lymphocyte (CTL) responses, immune-mediated tissue damage, viral persistence, and immune memory.

One major theme of this work was the regulation of immune responses through apoptosis . Ray and colleagues demonstrated that virus-induced lymphocyte apoptosis was not simply a mechanism for terminating immune responses, but a critical regulatory process that could prevent immunopathology while simultaneously shaping long-term immunity. These studies contributed to a broader understanding of immune contraction and homeostasis.

Ray also explored how viral infection alters antigen presentation, MHC class I expression, and susceptibility to immune-mediated killing (9). These mechanistic studies provided insight into how viruses evade immune responses and how immune pressure shapes viral evolution.

Perhaps, Ray’s most influential conceptual contribution was the systematic articulation of heterologous immunity (2)—the phenomenon by which immune responses to one pathogen influence responses to unrelated pathogens encountered later. Through elegant experimental work, Ray demonstrated that immunological memory is neither static nor uniformly protective (1, 2).

His laboratory showed that cross-reactive T cell populations could dominate responses to secondary infections, sometimes enhancing protection but often exacerbating immunopathology. These findings challenged traditional views of memory as a stable, beneficial archive and instead framed immune memory as a dynamic, competitive network.

Closely related was Ray’s work on memory attrition. He demonstrated that memory T cell populations could be eroded by subsequent infections through mechanisms independent of cognate antigen recognition (1). This insight had far-reaching implications for vaccine design, transplantation tolerance, and understanding immune aging.

Together, these studies reshaped thinking about immune history, emphasizing that each infection leaves an imprint that alters future immune responses in complex and sometimes unpredictable ways.

Ray was deeply committed to service within the scientific community. He served on the editorial boards of numerous journals, including long-standing service to the *Journal of Virology*. From 1997 to 2007, he served as an editor for JV, a role in which he was widely respected for his intellectual rigor, fairness, and insistence on mechanistic clarity.

He was also active in professional societies, including the American Society for Virology, the American Association of Immunologists, and the American Society for Microbiology. His service on NIH and American Cancer Society study sections allowed him to shape national research priorities over multiple decades.

Ray’s contributions were recognized through numerous honors, including an NIH MERIT Award, election as a Fellow of the American Association for the Advancement of Science, election to the American Academy of Microbiology, and multiple Outstanding Faculty Awards from the UMMS Graduate School of Biomedical Sciences.

If Ray’s scientific contributions defined his intellectual legacy, his mentorship defined his human one. Over the course of his career, he trained dozens of graduate students and postdoctoral fellows, many of whom went on to successful careers in academia, industry, biotechnology, and government research.

Former trainees consistently recall Ray’s distinctive mentoring philosophy. He encouraged independence, often remarking that he would give trainees “a rope long enough so that I can drag you in if I need to.” This approach fostered creativity, resilience, and intellectual ownership. Trainees were expected to think deeply, defend their ideas, and take responsibility for their science.

**Fig 1:**
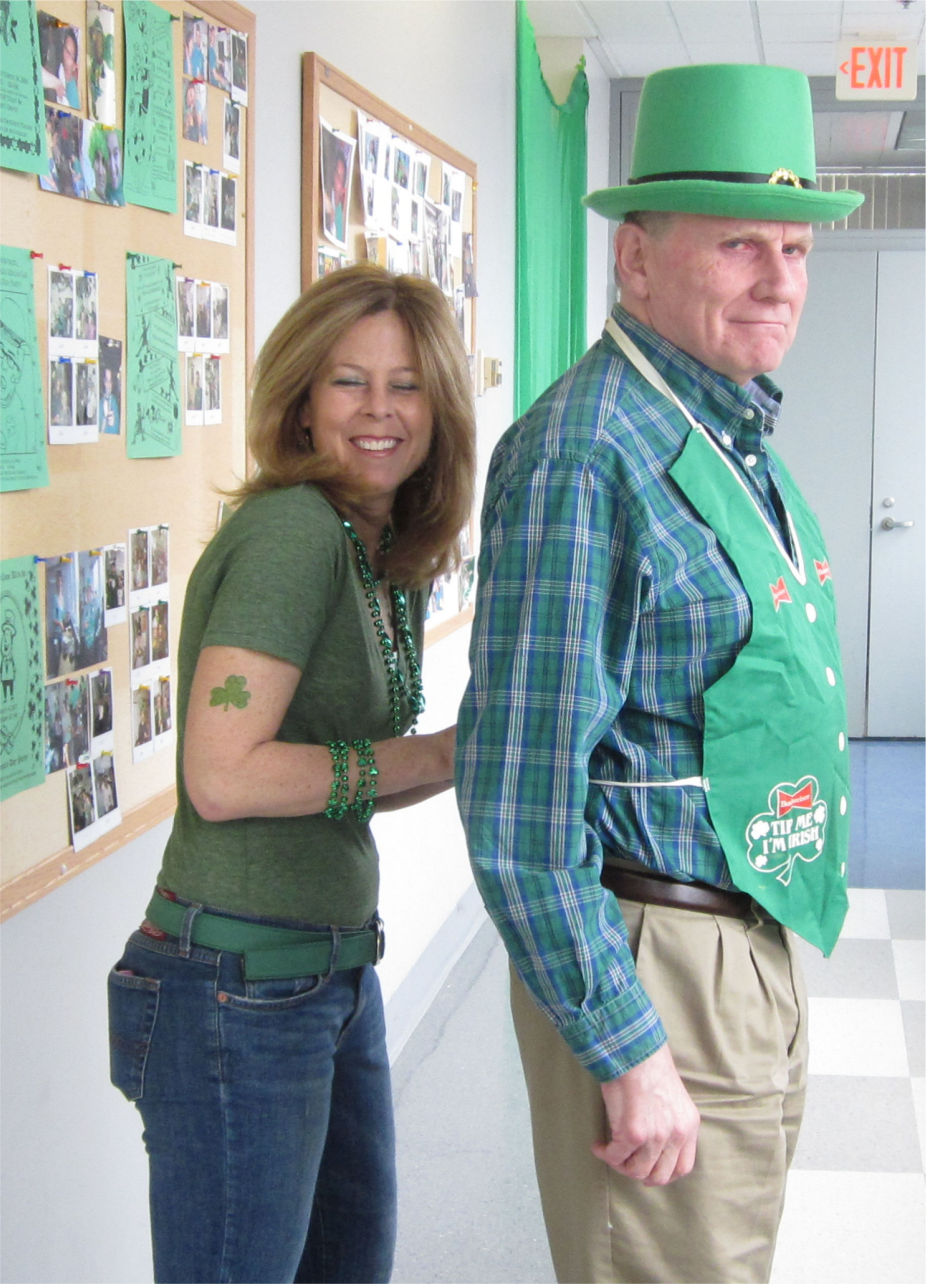
Carey helping Ray get ready for the St. Patrick’s Day party.

At the same time, Ray created a laboratory culture that emphasized community and continuity. Annual gatherings—most famously the Welsh Lab’s St. Patrick’s Day celebrations (Fig. 1)—became traditions that reinforced lab identity and alumni connections. These events were remembered not merely as social occasions, but as expressions of Ray’s belief that science is a collective enterprise sustained by shared experiences.

As former trainees noted, many of whom later became faculty members or industry leaders, Ray often joked, with characteristic humor, “Your job is to make me famous!” The remark captured both his confidence and his recognition that scientific legacy is carried forward by people, not publications alone.

In addition to his research and mentorship, Ray was a dedicated educator. He taught extensively in medical and graduate curricula, coordinating and lecturing in virology and immunology courses for decades. His lectures were known for their conceptual clarity, historical perspective, and willingness to confront unresolved questions.

Ray’s service to UMMS included extensive committee work, leadership of training programs, and long-standing involvement in biosafety and tenure committees. These roles reflected his commitment to sustaining institutional structures that support rigorous and ethical science.

In 2015, as his eyesight deteriorated, Ray made the decision to retire. His retirement was marked by a “Last Lecture” in 2019 that drew an extraordinary response from his scientific family. More than 90% of his current and former trainees attended in person or joined remotely. The event was both a celebration of his scientific contributions and a testament to the enduring bonds he had forged.

Dr. Raymond M. Welsh, Jr. passed away in 2024. His death prompted a renewed sense of connection among his former trainees and colleagues. His loss brought together scientists from around the world, reconnecting members of the Welsh laboratory across generations.

These reflections emphasized not only Ray’s scientific impact, but also his humanity—his humor, generosity, and commitment to maintaining relationships long after formal training had ended. Whether at national meetings or informal gatherings, Ray made a point of sustaining connections, modeling a vision of scientific life grounded in community.

Raymond M. Welsh, Jr. was a scientist who reshaped how virologists and immunologists think about immune regulation, memory, and host–virus interactions (1, 2, 7, 10). His work challenged reductionist models and insisted on grappling with complexity (2). At the same time, he built a mentoring culture that empowered generations of scientists to think independently and act responsibly.

His legacy endures in a vast and influential body of work, in the laboratories and institutions shaped by his trainees, and in the intellectual frameworks that continue to guide research in viral immunology. For the *Journal of Virology* community, Ray Welsh remains not only a pioneering scientist, but a defining figure in the journal’s history and the field it serves.

## References

[1] Selin LK , Welsh RM . 2004 . Plasticity of T cell memory responses to viruses . Immunity 20 : 5 – 16 . doi:10.1016/s1074-7613(03)00356-x 14738760 PMC7130098

[2] Welsh RM , Selin LK . 2002 . No one is naive: the significance of heterologous T-cell immunity . Nat Rev Immunol 2 : 417 – 426 . doi:10.1038/nri820 12093008

[3] Welsh RM . 1981 . Natural killer cells in virus infections . Curr Top Microbiol Immunol 92 : 83 – 106 . doi:10.1007/978-3-642-68069-4_6 6171387

[4] Biron CA , Welsh RM . 1982 . Activation and role of natural killer cells in virus infections . Med Microbiol Immunol 170 : 155 – 172 . doi:10.1007/BF02298196 6176843

[5] Welsh RM . 1986 . Regulation of virus infections by natural killer cells. A review . Nat Immun Cell Growth Regul 5 : 169 – 199 . 2430177

[6] Welsh RM . 1972 . Defective-interfering lymphocytic choriomeningitis virus Doctoral dissertation , University of Massachusetts Amherst

[7] Waggoner SN , Cornberg M , Selin LK , Welsh RM . 2012 . Natural killer cells act as rheostats modulating antiviral T cells . Nature 481 : 394 – 398 . doi:10.1038/nature10624 PMC3539796 22101430

[8] Welsh RM . 2007 . Lymphocytic choriomeningitis virus: general features . *In* Encyclopedia of virology . Elsevier .

[9] Zinkernagel RM , Welsh RM . 1976 . H-2 compatibility requirement for virus-specific T-cell–mediated effector functions in vivo . J Immunol 117 : 1495 – 1502 . doi:10.4049/jimmunol.117.5_Part_1.1495 794412

[10] Welsh RM , Bahl K , Marshall HD , Urban SL . 2012 . Type 1 interferons and antiviral CD8 T-cell responses . PLoS Pathog 8 : e1002352 . doi:10.1371/journal.ppat.1002352 22241987 PMC3252364

